# STAG2 regulates polycomb and differentiation in urothelial precursors and bladder cancer

**DOI:** 10.1371/journal.pone.0333128

**Published:** 2025-10-15

**Authors:** Youngrok Park, Wanying Xu, Tianyi Yang, Victoria Hill, Xiaoyuan He, Lisa Sadzewicz, Luke Tallon, Jung-Sik Kim, Fulai Jin, Todd Waldman

**Affiliations:** 1 Department of Oncology, Lombardi Comprehensive Cancer Center, Georgetown University School of Medicine, Washington, District of Columbia, United States of America; 2 Tumor Biology Training Program, Georgetown University School of Medicine, Washington, District of Columbia, United States of America; 3 The Biomedical Sciences Training Program, School of Medicine, Case Western Reserve University, Cleveland, Ohio, United States of America; 4 Department of Genetics and Genome Sciences, Case Comprehensive Cancer Center, Case Western Reserve School of Medicine, Cleveland, Ohio, United States of America; 5 Institute for Genome Sciences, University of Maryland, Baltimore, Maryland, United States of America; OMICS, PERU

## Abstract

The STAG2 tumor suppressor gene is commonly inactivated by mutations in a wide range of common cancer types. STAG2 encodes a component of the cohesin complex, which controls sister chromatid cohesion and 3D genome organization. In bladder cancer, STAG2 mutations are most common in the earliest low-grade lesions, suggesting that mutational inactivation of STAG2 may be an initiating event. To provide insight into the mechanisms of STAG2 tumor suppression in bladder cancer, siRNA and shRNA were used to knock down STAG2 in several different human non-neoplastic bladder cancer precursor cell lines. Gene editing was used to generate cultured human cancer cell lines that differ only in the presence or absence of bladder-cancer derived STAG2 mutations. These systems were interrogated using RNA-seq, Western blot, and qRT-PCR before and after induced differentiation. We find that inactivation of STAG2 in bladder cancer cells and in bladder epithelial precursor cells resulted in concomitant inactivation of the H3K27me3 Polycomb chromatin mark. Inactivation of STAG2 also attenuated induced differentiation of bladder epithelial precursor cells. STAG2 and other components of cohesin were upregulated during this differentiation process. This study provides new insights into the role of STAG2 in the pathogenesis of bladder cancer, demonstrating roles for STAG2 in the regulation of Polycomb-mediated epigenetic regulation and in the differentiation of bladder epithelial precursor cells.

## Introduction

Bladder cancer is the sixth most common cancer in the United States, with an annual incidence of approximately 80,000 cases [1]. The majority of bladder cancers are classified as urothelial carcinomas, originating from the epithelial cells lining the bladder wall. These tumors are broadly categorized into two subtypes: non-muscle-invasive bladder cancer (NMIBC), when the tumor remains confined to the bladder’s epithelial lining, and muscle-invasive bladder cancer (MIBC), when the tumor has penetrated the surrounding detrusor muscle [2,3].

Emerging research has highlighted the critical role of the cohesin complex in bladder cancer pathogenesis. Cohesin is a ring-like chromatin-bound protein complex that ensures proper chromatid segregation during cell division and regulates genome organization, gene expression, and DNA repair [4]. Inactivating mutations in genes encoding components of the cohesin complex are found in numerous human cancers, with STAG2 as by far the most commonly mutated subunit [5–7].

Truncating mutations of STAG2 account for over 50% of all cohesin gene mutations in cancer and are more common in bladder cancer than in any other cancer type. Approximately 35–40% of NMIBC tumors harbor STAG2 truncating mutations [8–10]. Interestingly, STAG2 mutations are much more common in the earliest bladder cancers (NMIBCs) than in late-stage bladder cancers (MIBCs). This finding revealed that NMIBCs caused by STAG2 mutations are less likely to progress to MIBC than NMIBCs caused by mutations of other bladder cancer tumor suppressors. As such, STAG2 mutational status may be useful as a biomarker for prediction of progression in NMIBC [11,12]. Despite the prevalence of STAG2 mutations in bladder cancer, the mechanism(s) through which STAG2 mutations drive its pathogenesis remain poorly understood.

Recent studies have suggested that STAG2 mutations may influence bladder cancer pathogenesis by disrupting key epigenetic regulatory pathways. Notably, cohesin has been shown to physically and functionally interact with the Polycomb Repressive Complex (PRC), a group of chromatin-modifying proteins that mediate transcriptional repression through histone methylation [13–17]. The PRC2 complex, which catalyzes the trimethylation of histone H3 at lysine 27 (H3K27me3), plays a critical role in gene silencing and is implicated in multiple cancers, including in bladder cancer (examples in [18,19,20]).

In addition to its role in epigenetic regulation, STAG2 has been implicated in controlling differentiation processes in various cell types [21–26]. In bladder cancer, STAG2 loss has been shown to disrupt urothelial differentiation, leading to the aberrant expression of basal and luminal cell markers [27]. This suggests that STAG2 mutations may lead to a defective differentiation program in bladder epithelial cells, increasing their susceptibility to malignant transformation. Taken together, these findings suggest that STAG2 mutations contribute to bladder cancer pathogenesis by altering both chromatin organization and differentiation processes.

In this study, we investigate the mechanistic role of STAG2 in bladder cancer, with a specific focus on its involvement in uroepithelial differentiation and the regulation of Polycomb-mediated epigenetic silencing. By elucidating the molecular consequences of STAG2 inactivation, we uncover novel insights into bladder cancer pathogenesis and identify potential therapeutic targets.

## Materials and methods

### Tissue culture

HBLAK cells and primary bladder epithelial (BdEC) cells were obtained from CellnTec and the American Type Culture Collection (ATCC), respectively. Both HBLAK and BdEC cells were cultured in CnT Prime Media (CellnTec), and the culture media was changed every 3 days. VM-CUB-3 cells were obtained from ATCC and cultured with DMEM (Life Technologies) containing 10% Fetal Bovine Serum (FBS, Sigma) and 1% penicillin/streptomycin (Life Technologies) at 37°C in 5% CO2. For terminal differentiation, HBLAK cells were treated at 60–70% confluence with 2mM calcium in Keratinocyte Serum Free Media (KSFM, Life Technologies) supplemented with 5% FBS, and BdEC cells were treated at 60–70% confluence with PD153035 (Merck) and troglitazone (Cayman) in KSFM with 5% FBS.

### RNA-seq

Total RNA was isolated using the RNeasy Mini Kit (Qiagen). RNA-seq libraries were prepared using the NEB Next Ultra II RNA Library Prep Kit (NEB) and sequenced on an Illumina NovaSeq 6000 instrument. RNA-seq data were aligned to the human reference genome (hg19) by using HISAT2 [28], and read counts for each gene were called using featureCounts [29]. The statistical tests for RNA-seq data, normalizing raw data and identifying differentially expressed genes in this study were derived from a statistical model derived from DESeq2 [30]. All RNA-seq data and lists of differentially expressed genes are provided in S1 Table.

### qRT-PCR

Quantitative reverse transcription-PCR (qRT-PCR) was performed in an StepOnePlus Real-Time PCR Thermocycler (Applied Biosystems) using TaqMan assays (Thermo Fisher) and the Superscript III Platinum One Step qRT-PCR System (Invitrogen), according to the manufacturers’ specifications. Catalog numbers and amplicon size for each assay is provided in S2 Table. Relative gene expression levels were calculated using the 2^−Δ(ΔCT)^ method, normalizing to the expression of the GAPDH housekeeping gene. All assays were performed at least in triplicate and errors bars represent standard deviations.

### shRNA and siRNA-mediated STAG2 depletion

Identification and validation of lentiviral STAG2 shRNA constructs have been described previously [5]. Lentiviruses were packaged by cotransfection of 293T cells (ATCC) with lentiviral helper plasmids pHR′CMV8.2ΔR and pCMV-VSV-G as previously described. Virus-containing conditioned medium was harvested 48 h after transfection, filtered and used to infect recipient cells in the presence of 8 μg/ml polybrene. Infected cells were selected with 2 μg/ml puromycin until all mock-infected cells were dead and were then maintained in puromycin. For siRNAs, cells were transfected with Silencer Select siRNAs (Ambion) using RNAiMax (Thermo Fisher) according to the manufacturer’s instructions.

### Correction of mutant STAG2 by gene editing in VM-CUB-3 cells

An AAV-based gene editing vector for correction of the STAG2 splice acceptor mutation in VM-CUB-3 cells was designed (for schematic, see S4 Fig). The left homology arm (LHA; ~ 1 kb) contains wild-type genomic sequence corresponding to intron 5, exon 6, and the first 200 nucleotides of intron 6. The right homology arm (RHA; ~ 1 kb) contains wild-type genomic sequence corresponding to intron 6. Homology arms were synthesized by Genscript and cloned into pAAV-SEPT, an AAV-based gene editing acceptor vector we previously reported [31] in which polylinkers for the cloning of LHAs and RHAs flank a promoterless splice-acceptor-IRES-neo^R^ gene.

This STAG2 gene editing vector was then packaged into AAV-2 (AAV2/2) virions by co-transfection into HEK293T cells with helper plasmids pAAC- RC and pHELPER using X-tremeGENE 9 (Roche Diagnostics) according to manufacturers’ instructions. Two days after transfection, media was aspirated, and cell monolayers were scraped into 1 mL PBS and subjected to four cycles of freeze/thaw. The lysate was then clarified by centrifugation at 12,000 rpm for 10 min in a benchtop microfuge to remove cell debris, and the virus-containing supernatant was aliquoted and stored at −80°C.

VM-CUB-3 recipient cells were then transduced with 200μl virus overnight in a T25 flask and plated out at limiting dilution into 96-well plates in media containing 0.5 mg/ml G418. Genomic DNA was extracted from G418-resistant colonies and tested for the presence of homologous integration of the targeting vector using a primer pair specific for the targeted allele. DNA sequencing confirmed that the mutant allele of STAG2 had been corrected. Cells confirmed to have undergone mutation correction were expanded, infected with cre-expressing adenovirus overnight, and plated at limiting dilution into 96-well plates. Single colonies were expanded and tested for G418 sensitivity. Sensitive cells were expanded and tested for re-expression of STAG2 protein by Western blot.

### Western blot

Protein lysates were dissolved in LDS sample buffer, boiled for 5 min, and separated by SDS-PAGE. Proteins were transferred to polyvinylidene difluoride membranes, which were then probed with a 1:1000 dilution of primary antibodies overnight rotating at 4°C. After incubation with horseradish peroxidase–conjugated secondary antibodies (Cell Signaling) rotating for 1 h at room temperature, membranes were developed with SuperSignal West Pico PLUS chemiluminescent substrate (Pierce) and imaged using a myECL imager (Pierce). See S1 Fig for original Western blots.

### Antibodies

Primary antibodies for immunoblotting were STAG2 (sc-81852), from Santa Cruz Biotechnology; GAPDH (2118), histone H3 (14269), H3K27me3 (C36B11), and EZH2 (5246) from Cell Signaling Technologies; and RAD21 (A300-080), SMC1A (A300-055), SMC3 (A300-060), and STAG1 (A302-579) from Bethyl Laboratories.

## Results

### A bladder cancer-derived STAG2 mutation generates a Polycomb expression signature in human cells

**Fig 1:**
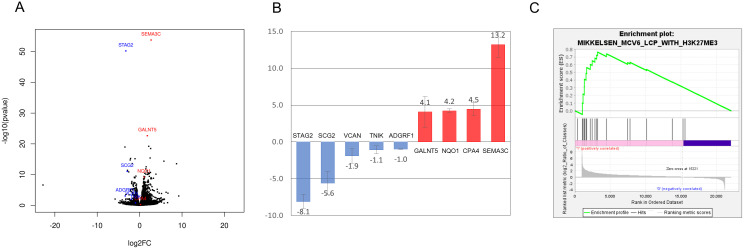
RNA-seq analysis of gene expression in isogenic STAG2-mutant cells. (A) Volcano plot of differentially expressed genes in S97X mutant cells (P < 0.01 and |logFC| > 1). The entire gene list is presented in S1 Table. (B) qRT-PCR confirmation of a subset of the differentially expressed genes, individually denoted in color in A. Each assay was performed at least in triplicate, and error bars represent standard deviation. (C) GSEA analysis of STAG2-dependent gene expression reveals a polycomb/H3K27me3 gene expression signature.

To identify putative effectors of STAG2 tumor suppression, RNA-seq was performed on a previously reported isogenic set of human cancer cells that differ only in the presence or absence of a bladder cancer-derived STAG2 mutation introduced by gene editing (S97X; Fig 1A) [32]. RNA-seq was performed on biological triplicates of parental cells and biological duplicates of isogenic STAG2 S97X gene edited-derivatives (282–422 million read pairs/sample; data summary in S1 Table). There were 319 upregulated genes and 81 downregulated genes at P < 0.01 and |logFC| > 1; see S1 Table for a complete gene list and S2 Fig for Gene Ontology (GO) analysis of the differentially expressed genes. A subset of the most robustly differentially expressed genes were confirmed by qRT-PCR (Fig 1B; assay details in S2 Table).

Gene Set Enrichment Analysis (GSEA) was then performed to identify potential expression signatures of the S97X bladder cancer-derived truncation mutation in the STAG2 gene [33]. See S3 Table and S3 Fig for all GSEA signatures meeting a threshold of FDR < 0.05. Among the most significant signatures was “MIKKELSEN MCV6 LCP WITH H3K27ME3” (FDR = 0.012), derived from genes regulated by H3K27me3 Polycomb chromatin marks during reprogramming of somatic cells into iPS cells (Fig 1C) [34]. This finding suggested that STAG2 mutations may drive the pathogenesis of bladder cancer by altering Polycomb-mediated histone trimethylation and bladder cell differentiation. Also interestingly, four of the twelve STAG2-mutant GSEA signatures are related to interferon response; this finding will be further described elsewhere.

### STAG2 regulates Polycomb signaling in culture human bladder epithelial cells

**Fig 2:**
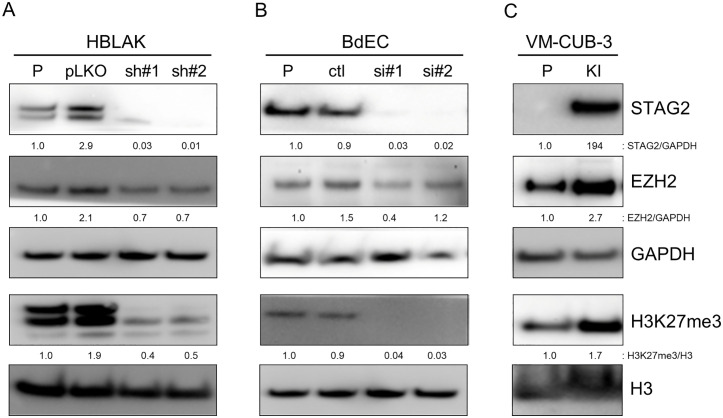
STAG2 regulates H3K27me3 levels in uroepithelial cells. shRNA/siRNA-mediated inactivation of STAG2 in non-cancerous HBLAK (A) and BdEC cells (B) results in decreased level of H3K27me3 expression. In VM-CUB-3 human bladder cancer cells (C), correction of the endogenous mutated allele of STAG2 by gene editing results in re-expression of the STAG2 protein and increased levels of H3K27me3; “P” denotes parental cells, “pLKO” denotes cells infected with lentiviral empty vector; “sh#1” and “sh#2” denotes cells infected with one of two different STAG2 shRNAs; “ctl” denotes cells transfected with a scrambled control siRNA; “si#1” and “si#2” denotes cells transfected with one of two different STAG2 siRNAs; KI = knock-in.

We next wanted to determine if STAG2 mutations alter Polycomb signaling in human bladder cells. To do this, we used HBLAK (CellnTEC) and BdEC (ATCC) cells [35]. BdEC are primary human bladder epithelial cells, and HBLAK is a non-tumorigenic, spontaneously transformed human bladder epithelial cell line. Both cells have wild-type STAG2 genes. To determine the effect of STAG2 inactivation on Polycomb, Western blot was performed on RIPA lysates from parental cells and derivatives in which STAG2 had been knocked down by either siRNA or shRNA using antibodies to STAG2, H3K27me3, and EZH2, the catalytic component of the PRC2 complex. Stable lentiviral shRNA was used in HBLAK cells since they are an immortalized cell line suitable for long term passage and selection, whereas transient transfection of siRNA was used in BdEC cells since they are primary cells that quickly senesce. Depletion of STAG2 resulted in substantially decreased levels of the H3K27me3 Polycomb mark (Fig 2A,B).

### Correction by gene editing reveals that STAG2 regulates Polycomb signaling in human bladder cancer cells

Next, we sought to generate an experimental system allowing us to extend these experiments to human bladder cancer cells. To do this, gene editing was used to correct the endogenous, naturally occurring exon 6 A > G splice acceptor mutation of STAG2 in VM-CUB-3, a human bladder cancer cell line [8]. For the details of gene editing, see Materials and Methods and S4 Fig. Next, Western blot was performed to assess the effect of STAG2 correction on the H3K27me3 chromatin mark in VM-CUB-3 cells and their isogenic STAG2-corrected derivatives (Fig 2C). Mutation correction resulted in re-expression of wild-type STAG2 and enhanced expression of both the H3K27me3 mark and EZH2, the PRC2 catalytic subunit that generates it.

### STAG2 is required for the complete differentiation of bladder epithelial cells

**Fig 3:**
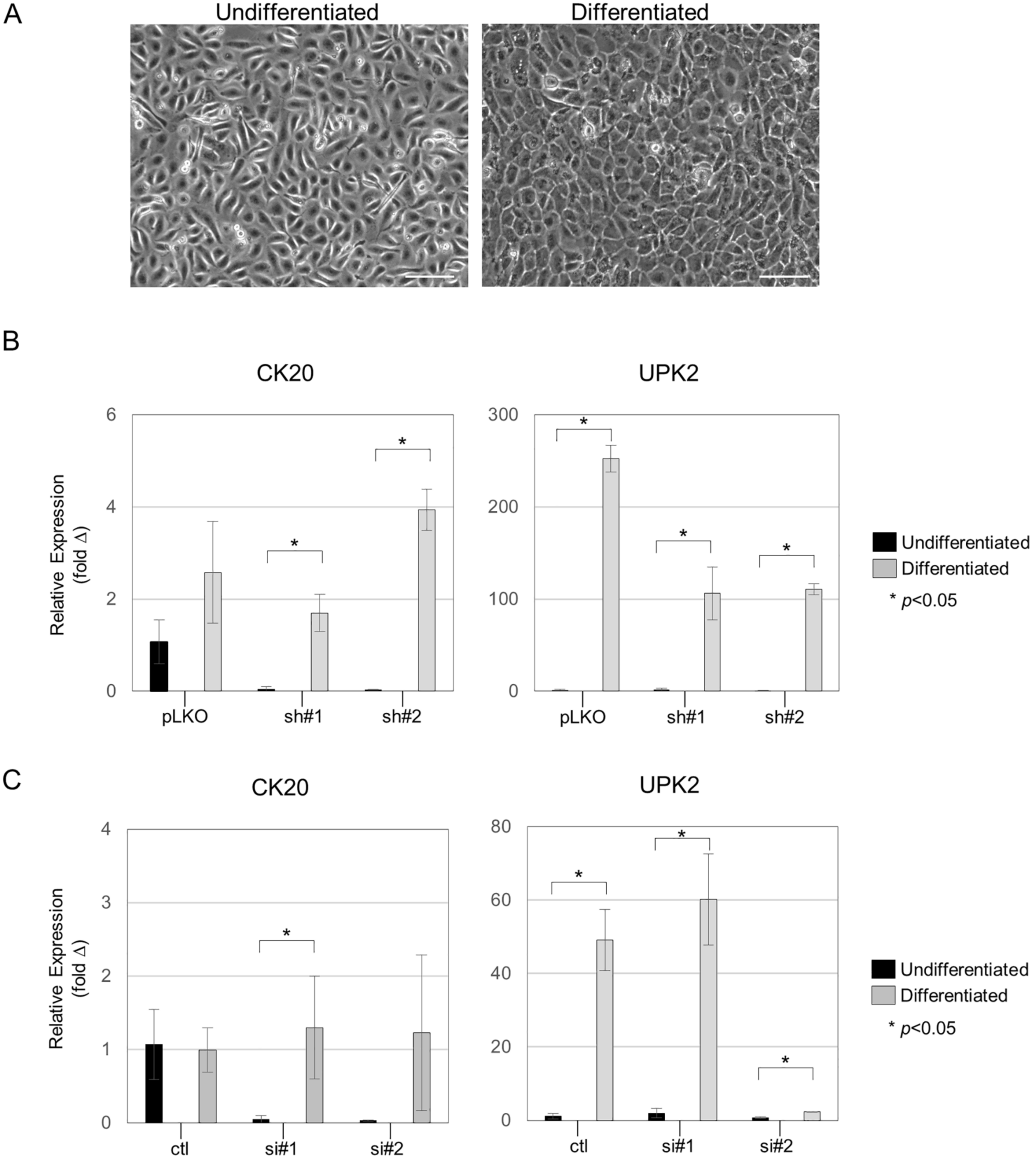
Effects of STAG2 inactivation on differentiation in HBLAK and BdEC cells. (A) Photomicrographs of HBLAK cells before and after induced differentiation. Scale bar = 100 um. (B, C) Expression of the urothelial cell terminal differentiation markers CK20 and UPK2 was measured by qRT-PCR in (B) HBLAK and (C) BdEC cells before and after induced differentiation. “pLKO” denotes cells infected with lentiviral empty vector; “sh#1” and “sh#2” denotes cells infected with one of two different STAG2 shRNAs; “ctl” denotes cells transfected with a scrambled control siRNA; “si#1” and “si#2” denotes cells transfected with one of two different STAG2 siRNAs.

We next evaluated the effect of STAG2 inactivation on the terminal differentiation of bladder epithelial cells. Both HBLAK and BdEC cells can be differentiated in vitro into terminal uroepithelial cells (“umbrella cells”) which express the urothelial-specific markers UPK2 and CK20. Isogenic sets of HBLAK and BdEC parental and STAG2 knock-down cells (shown in Fig 2) were differentiated as described in Materials and Methods (morphology changes shown in Fig 3A), and qRT-PCR for the CK20 and UPK2 differentiation markers was performed (Fig 3B; assay details in S2 Table). In both cell systems, differentiation resulted in a >100-fold increase in the expression of UPK2 and a 2–5 fold increase in CK20 (Fig 3B), increases that were partially dependent on the presence of STAG2.

### Components of the cohesin complex are upregulated in terminally differentiated HBLAK cells

**Fig 4:**
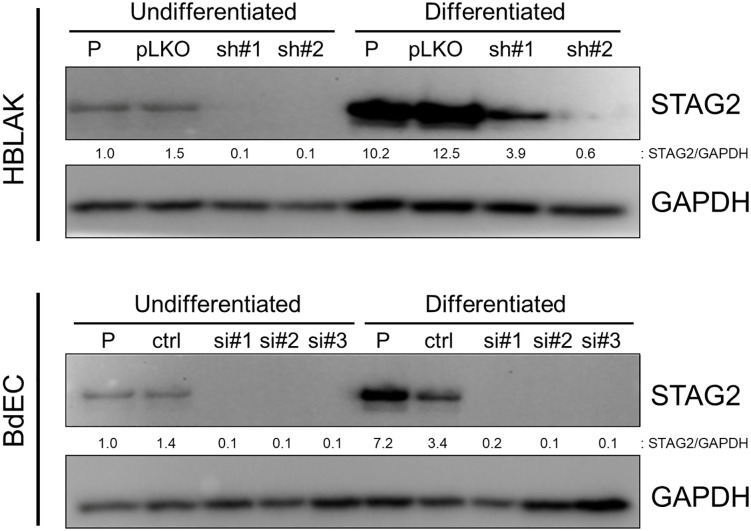
STAG2 upregulation in terminally differentiated HBLAK and BdEC cells. Western blot analysis of STAG2 protein in HBLAK cells (top) and BdEC cells (bottom) before and after induced differentiation. “P” denotes parental cells, “pLKO” denotes cells infected with lentiviral empty vector; “sh#1” and “sh#2” denotes cells infected with one of two different STAG2 shRNAs; “ctl” denotes cells transfected with a scrambled control siRNA; “si#1,” “si#2,” and “si#3” denote cells transfected with one of three different STAG2 siRNAs.

**Fig 5:**
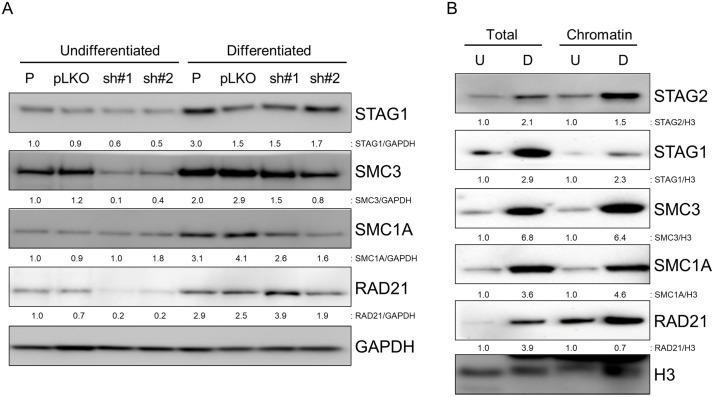
Various components of the cohesin complex are upregulated in terminally differentiated HBLAK cells. (A) Western blot analysis of cohesin proteins STAG1, SMC3, SMC1A, and RAD21 in parental HBLAK cells (P), and derivatives infected with either empty vector (pLKO) or two different STAG2 lentiviral shRNAs (sh#1, sh#2) before and after terminal differentiation withy CaCl_2_. (B) Western blot analysis of the same cohesin proteins in whole cell protein lysates and in chromatin fractions. “U” refers to undifferentiated cells, “D” refers to differentiated cells.

While testing the differentiation capacity of HBLAK cells, we unexpectedly observed a marked upregulation of STAG2 during terminal urothelial differentiation (Fig 4). To further investigate this, we repeated the differentiation process, then performed Western blot on protein lysates from the isogenic set of HBLAK cells and their Ca2 + -differentiated derivatives using antibodies against the other core cohesin subunit proteins – STAG1, SMC3, SMC1A, and RAD21. As shown in Fig 5A, the expression of each cohesin subunit increased after differentiation. However, the increase in STAG2 (Fig 4) was by far the greatest, suggesting that STAG2 may be a particularly important player during urothelial differentiation.

The cohesin complex is known to exist in two pools in the nucleus – either bound to chromatin, or in the soluble nuclear fraction. To determine which pool of cohesin was upregulated during bladder differentiation, we performed Western blot on chromatin and soluble nuclear fractions of HBLAK cells and their differentiated derivatives. As shown in Fig 5B, both the chromatin-bound and soluble components of the core cohesin subunits were upregulated after differentiation. Taken together, these findings suggest a role for cohesin – particularly its STAG2 subunit – in bladder cell differentiation.

## Discussion

In this study, we provide new insights into the role of STAG2 in bladder cancer, demonstrating its involvement in both Polycomb-mediated epigenetic regulation and bladder epithelial cell differentiation. By utilizing gene-editing approaches in human bladder cells and cancer models, we show that STAG2 mutations disrupt the normal epigenetic landscape by inhibiting Polycomb Repressive Complex 2 (PRC2) expression. These findings suggest a new avenue by which STAG2 mutations contribute to bladder cancer pathogenesis and offer potential new targets for therapeutic intervention.

Our RNA-seq analysis revealed a distinct gene expression signature linked to Polycomb-regulated H3K27me3 marks, indicating that STAG2 mutations may impact chromatin modifications essential for maintaining the differentiated state of bladder epithelial cells. This suggests that STAG2-mutant tumors may, in part, arise due to faulty chromatin silencing and differentiation programs. Moreover, the observed alteration of H3K27me3 levels in STAG2 knock-down cells points to a potential mechanistic link between cohesin dysfunction and Polycomb deregulation in bladder cancer.

It is arguably surprising that STAG2 inactivation would result in downregulation of a gene (EZH2) and pathway (Polycomb) whose overexpression and activation is generally associated with enhanced aggressiveness of cancer. However, based on previous studies in our lab and others, we know that the effect of STAG2 loss on Polycomb may be tumor-type specific; prior studies have shown that Polycomb is upregulated by STAG2 loss in brain cancer [17], but downregulated by STAG2 loss in Ewing sarcoma [20]. We believe that the finding reported here that Polycomb is downregulated by STAG2 loss in NMIBC could help explain why STAG2-mutant NMIBC tumors tend to be less aggressive than NMIBC tumors caused by mutations in other tumor suppressor genes [11,12].

Our findings also support the emerging concept of crosstalk between the cohesin and Polycomb complexes. In previous studies, cohesin has been shown to facilitate genome architecture and gene expression by stabilizing chromatin loops [36,37]. The interaction between cohesin and Polycomb complexes may thus be necessary for the proper organization of chromatin domains that repress oncogenic programs during differentiation. Our results extend this understanding, showing that STAG2 mutations lead to loss of the H3K27me3 mark and disrupt PRC2 function, both in normal bladder epithelial cells and bladder cancer cells. This dysregulation could help explain the de-differentiated and stem-cell-like characteristics observed in bladder tumors.

In addition to its role in epigenetic regulation, we also demonstrated that STAG2 appears to be involved in the complete terminal differentiation of bladder epithelial cells. This finding is consistent with prior reports linking STAG2 to differentiation control in other tissue types, including hematopoietic and epithelial systems.

Interestingly, we also discovered that STAG2 and other core cohesin components are upregulated during terminal differentiation of bladder epithelial cells, further suggesting that cohesin proteins may be important to this differentiation process. Both chromatin-bound and soluble cohesin components were increased during urothelial differentiation. That said, most of the findings in this study are STAG2-centric, and any implications for SMC3, SMC1A, RAD21, or other components of the cohesin complex are suggestive but not directly supported by experimental evidence.

The therapeutic implications of these findings are potentially significant. Our results suggest the hypothesis that targeting the cohesin-Polycomb axis may provide therapeutic benefit for patients with STAG2 mutations. Given the important role of STAG2 in regulating both differentiation and epigenetic silencing, therapeutic strategies that restore normal chromatin dynamics or target the altered differentiation programs in these tumors could be promising avenues for treatment.

In conclusion, our study identifies STAG2 as a player in the regulation of Polycomb-mediated gene repression and bladder epithelial differentiation. We believe that further research focusing on the specific mechanisms through which cohesin dysfunction alters Polycomb signaling and how this contributes to the malignant transformation of bladder epithelium may prove informative.

## Supporting information

S1 Fig Original western blot scans. (PDF)

S2 Fig GO analysis of STAG2-regulated gene expression. (PPTX)

S3 Fig GSEA analysis of STAG2-regulated gene expression. GSEA signatures meeting a threshold of FDR < 0.05 are shown.
(PPTX)

S4 Fig Strategy for STAG2 gene correction in VM-CUB-3 cells. (A) Schematic of STAG2 exon 5/intron 5/exon 6. (B) Gene editing strategy for correction of the STAG2 splice acceptor mutation in VM-CUB-3 cells. The location of the splice acceptor mutation in VM-CUB-3 cells is shown with a red asterix. An AAV-based gene editing vector was generated in which ~1 kb homology arms composed of wild-type STAG2 genomic sequence flank a FLOXed IRES-neo^R^ gene. After infection of VM-CUB-3 cells, a subset of neo^R^ clones have integrated the gene editing vector by homologous recombination, resulting in correction of the splice acceptor mutation. The FLOXed neoR gene in intron 6 is then removed by cre-recombination by infection with a cre-expressing adenovirus.
(PPTX)

S1 Table Gene expression analysis of STAG2 mutant isogenic cells by RNA-seq. (XLSX)

S2 Table Taqman assays used in this study. (DOCX)

S3 Table GSEA analysis of STAG2-regulated gene expression. GSEA signatures meeting a threshold of FDR < 0.05 are listed. “SIZE” is the number of genes contained in the gene set. “ES” is the Enrichment Score. “NES” is the Normalized Enrichment Score. “FDR” is the False Discovery Rate. “RANK AT MAX” is the position in the ranked gene list where the maximum ES is reached. “LEADING EDGE” is the subset of genes within a gene set that contributes most to the ES.
(XLSX)
