# Supplementary material for: STAG2 regulates polycomb and differentiation in urothelial precursors and bladder cancer
Source: PLoS One. 2025 Oct 15;20(10):e0333128. doi: 10.1371/journal.pone.0333128 (PMC12527211; doi:10.1371/journal.pone.0333128)
Supplement: S1 Fig — (PDF) [file pone.0333128.s001.pdf]

S1 Fig

Scans of Western Blots for Fig. 2A

(The region shown in the Figure is denoted by a red box)

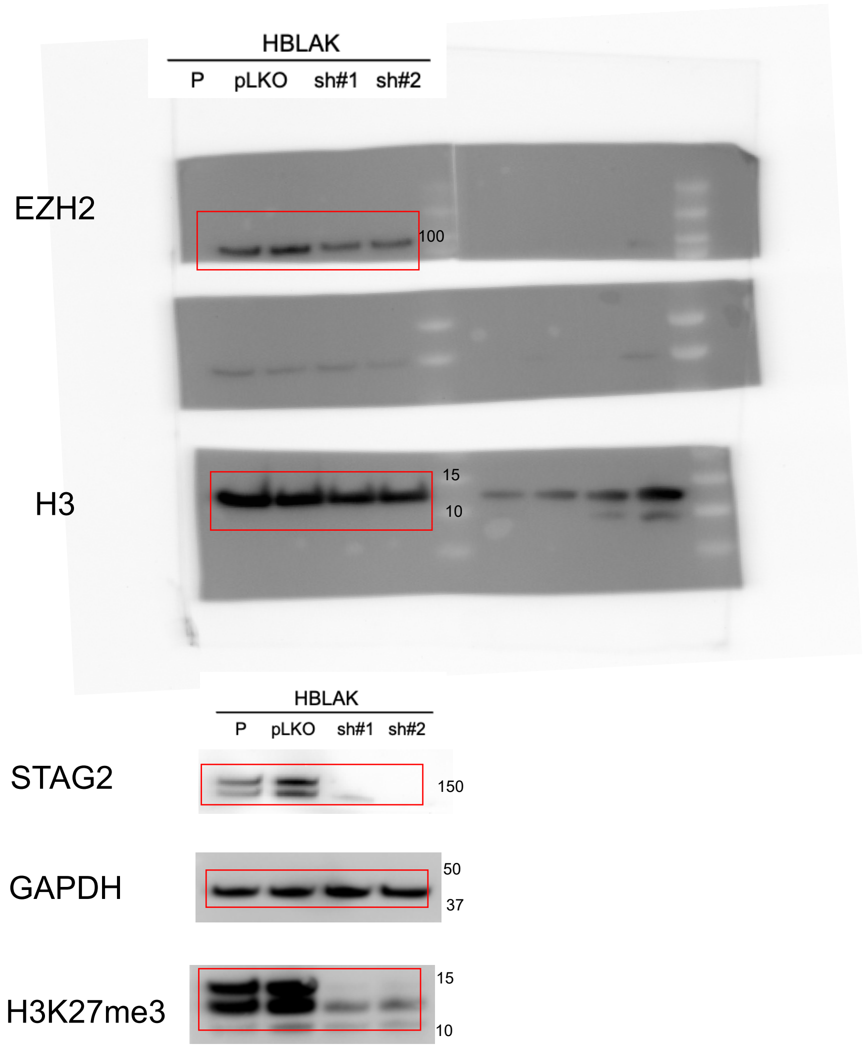

S1 Fig

Scans of Western Blots for Fig. 2B
(The region shown in the Figure is denoted by a red box)

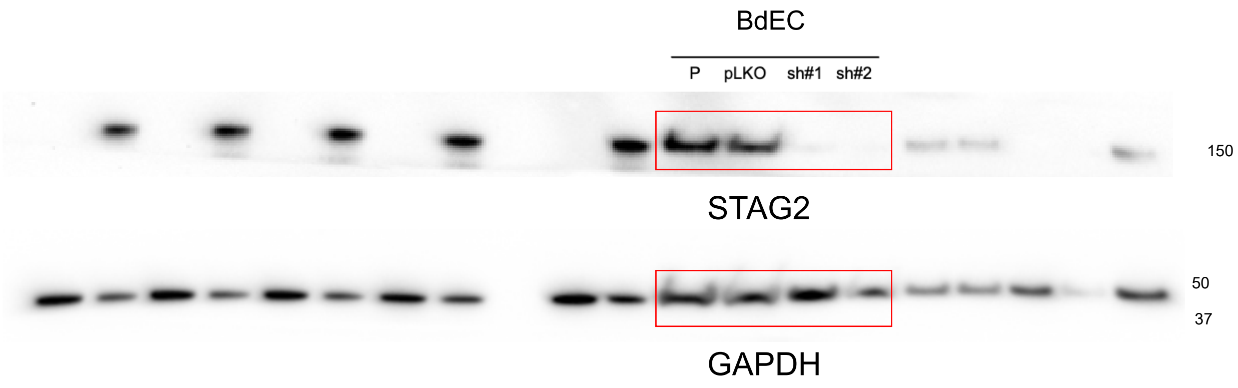

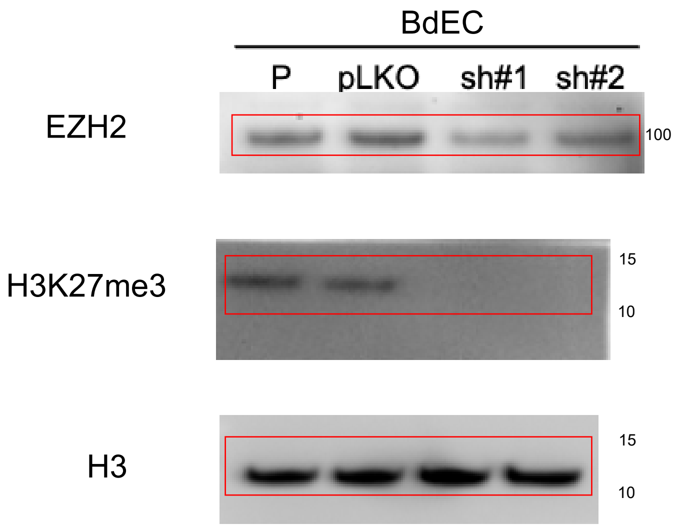

S1 Fig

Scans of Western Blots for Fig. 2C

(The region shown in the Figure is denoted by a red box)

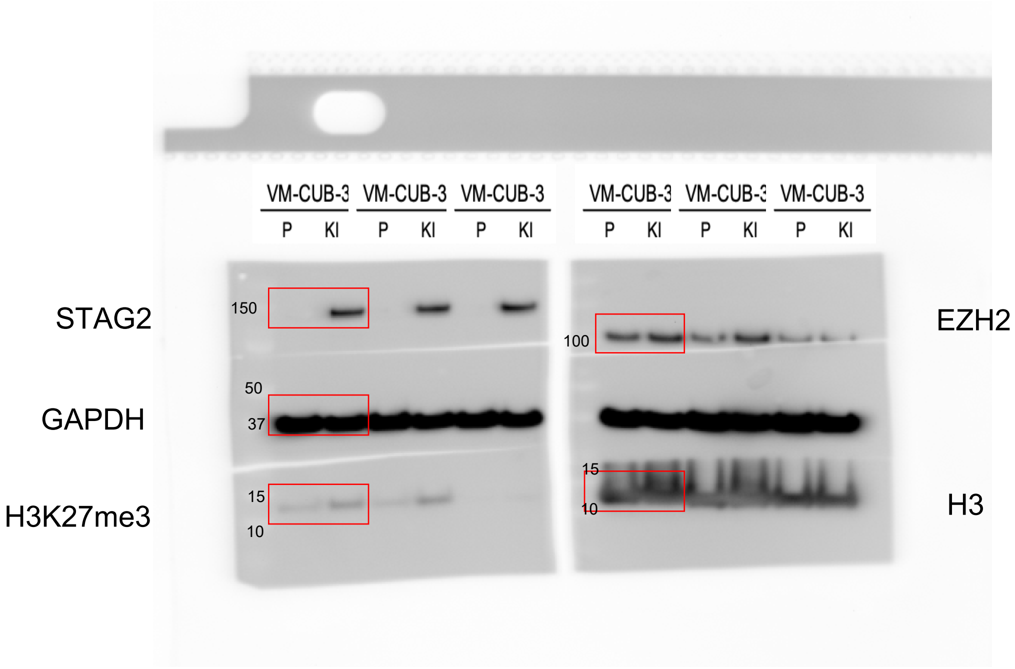

S1 Fig

Scans of Western Blots for Fig. 4

(The region shown in the Figure is denoted by a red box)

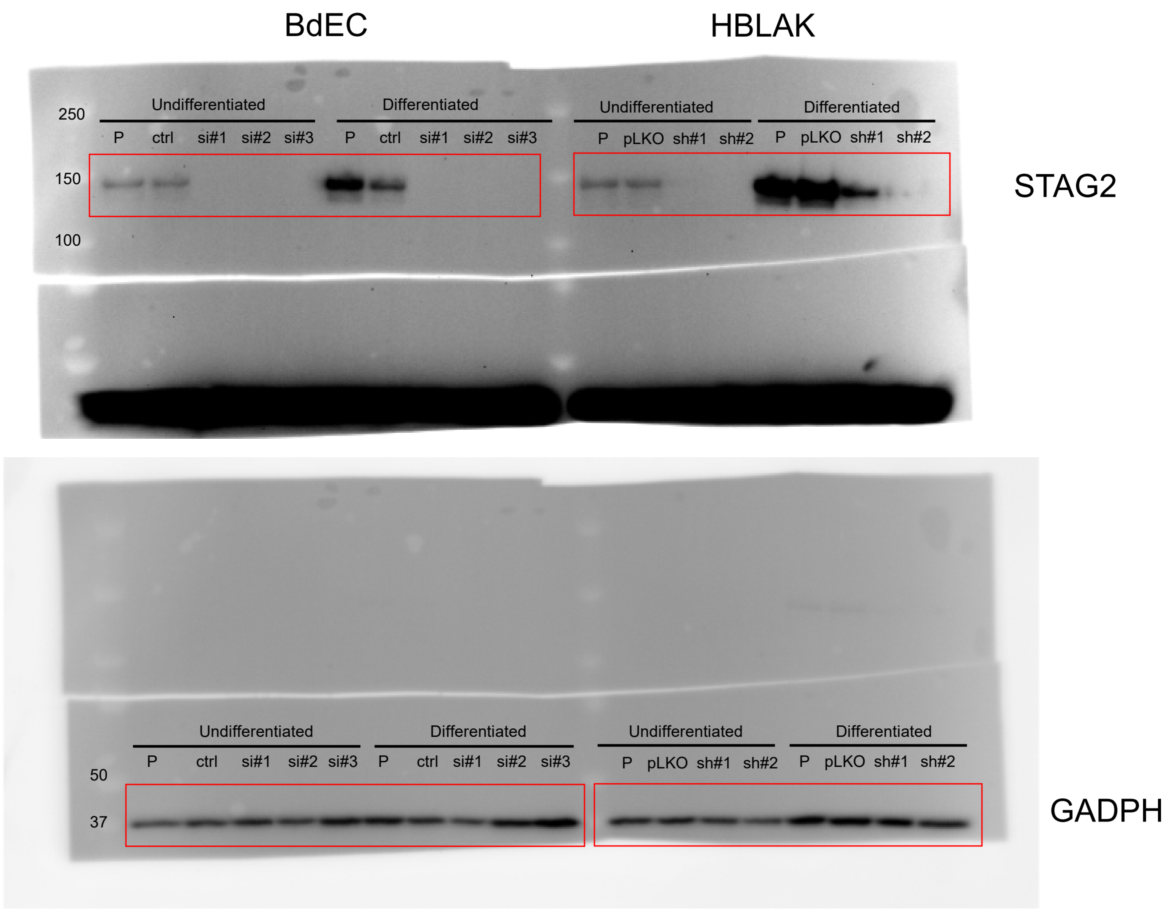

S1 Fig

Scans of Western Blots for Fig. 5A

(The region shown in the Figure is denoted by a red box)

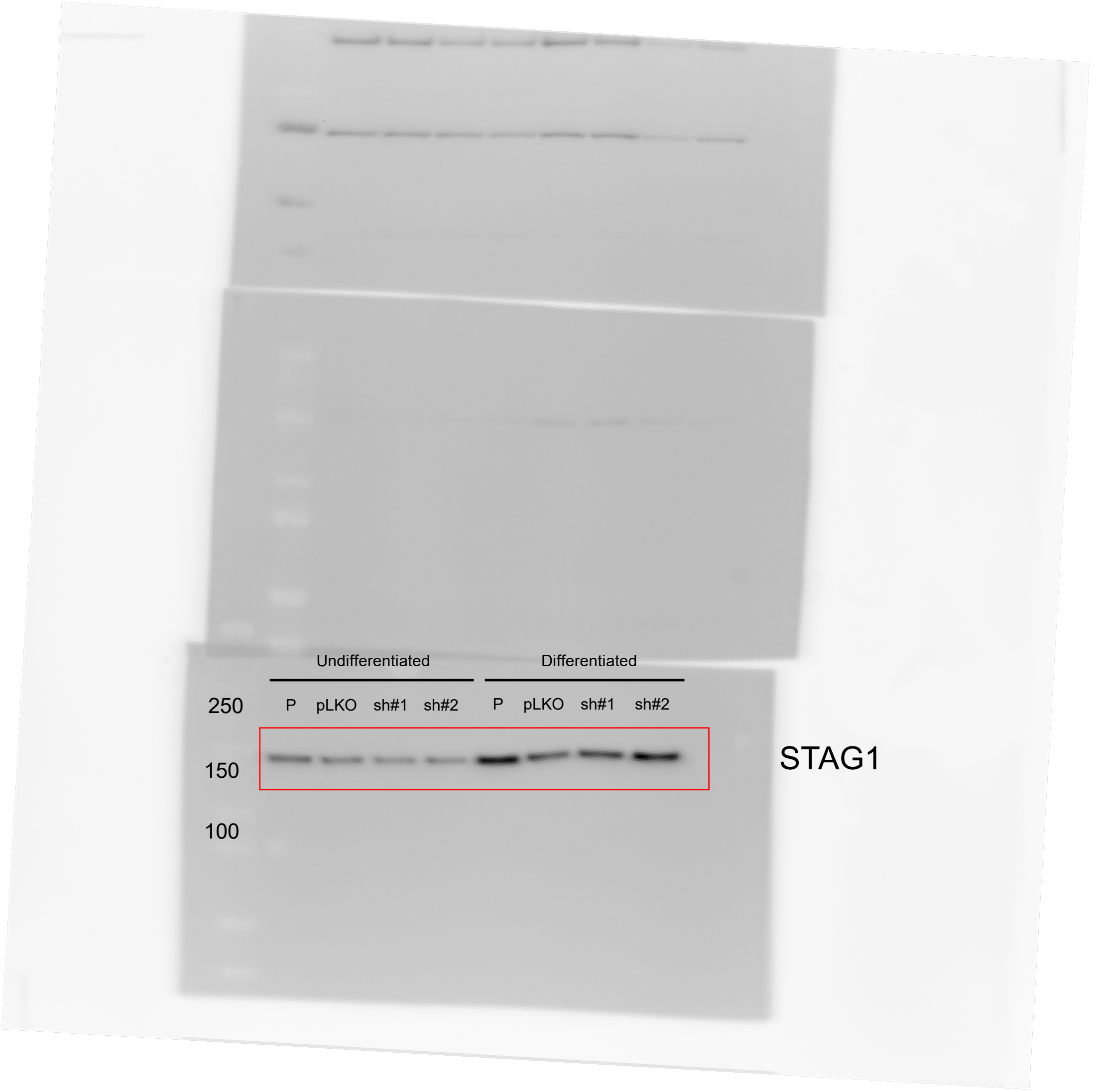

S1 Fig

Scans of Western Blots for Fig. 5A

(The region shown in the Figure is denoted by a red box)

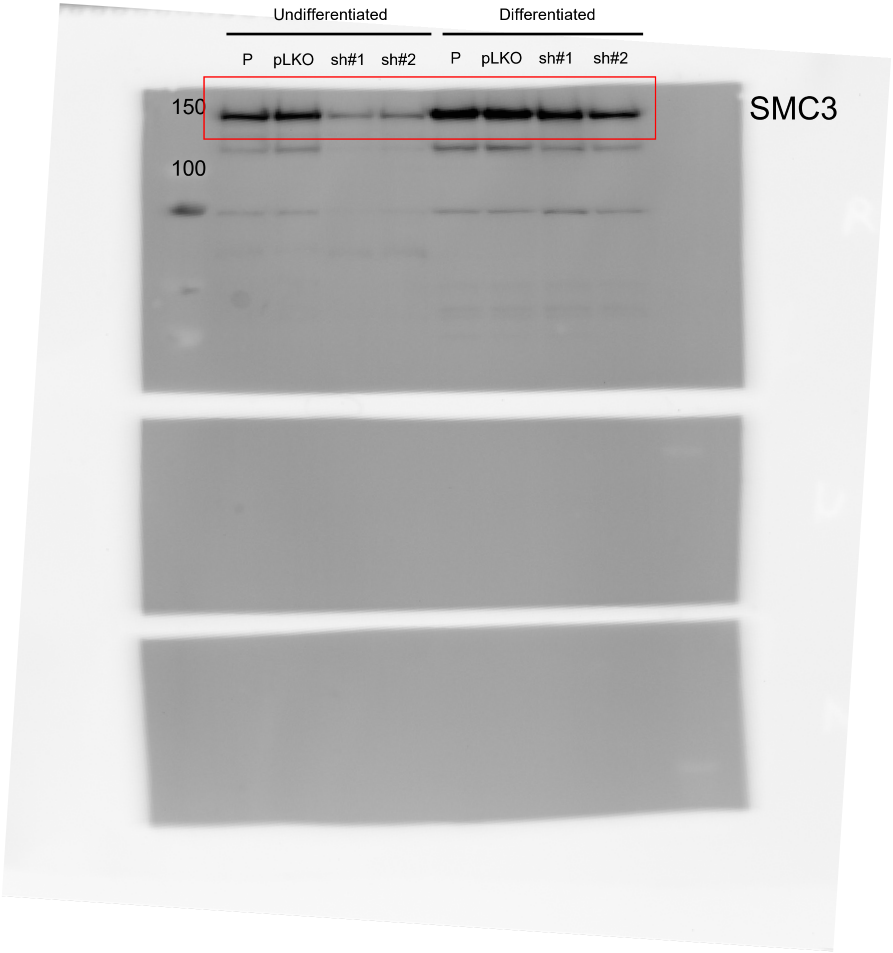

S1 Fig

Scans of Western Blots for Fig. 5A

(The region shown in the Figure is denoted by a red box)

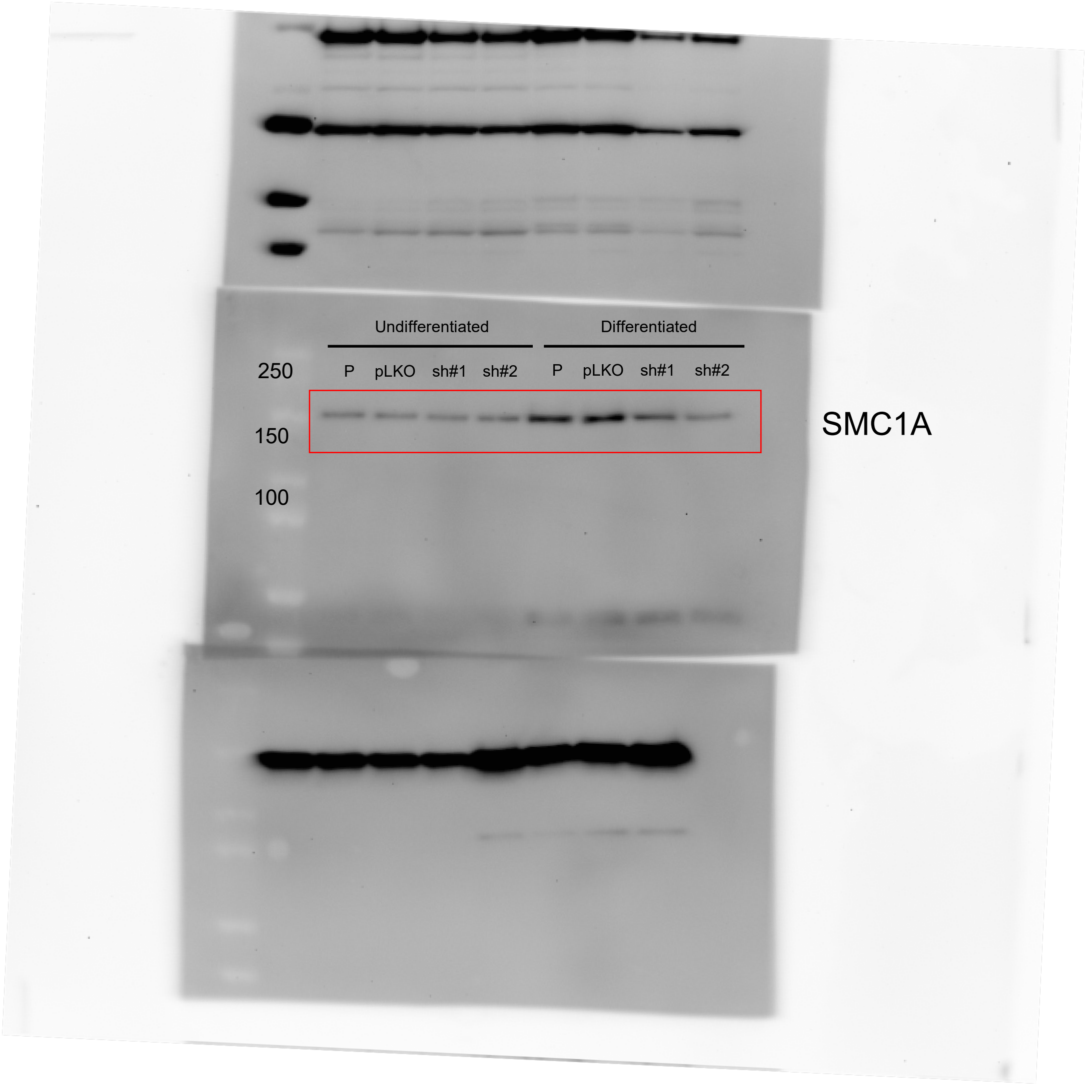

S1 Fig

Scans of Western Blots for Fig. 5A

(The region shown in the Figure is denoted by a red box)

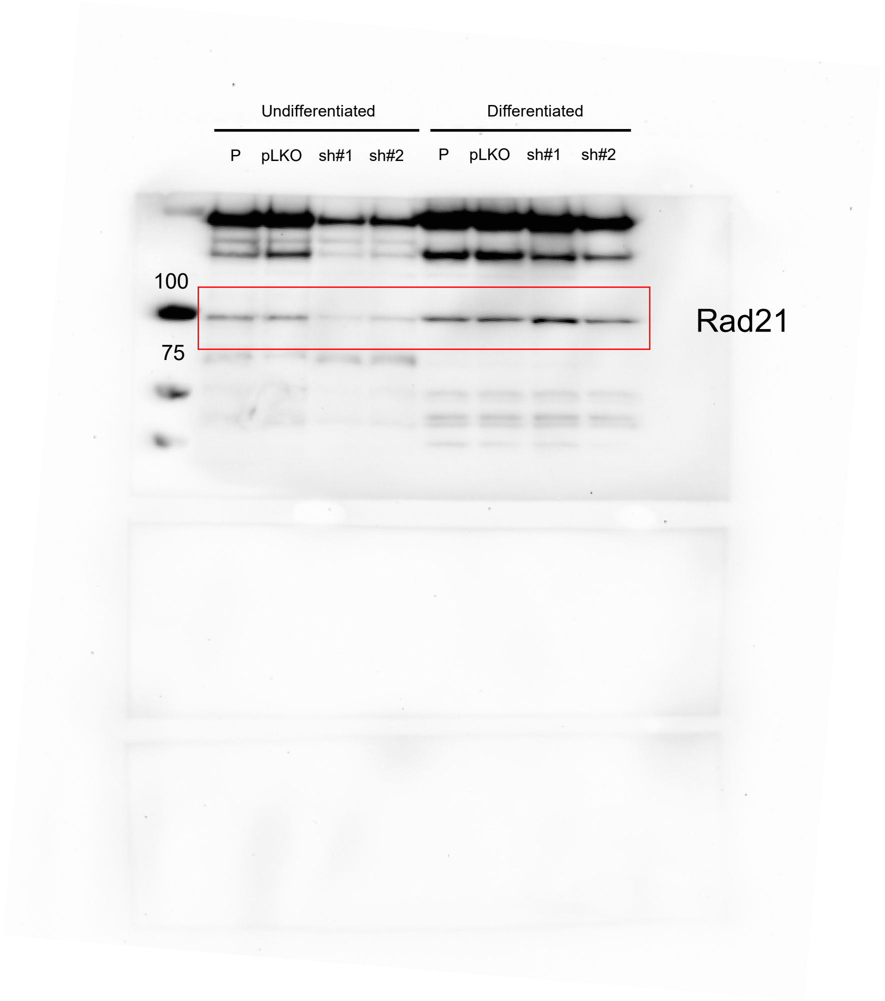

S1 Fig

Scans of Western Blots for Fig. 5A

(The region shown in the Figure is denoted by a red box)

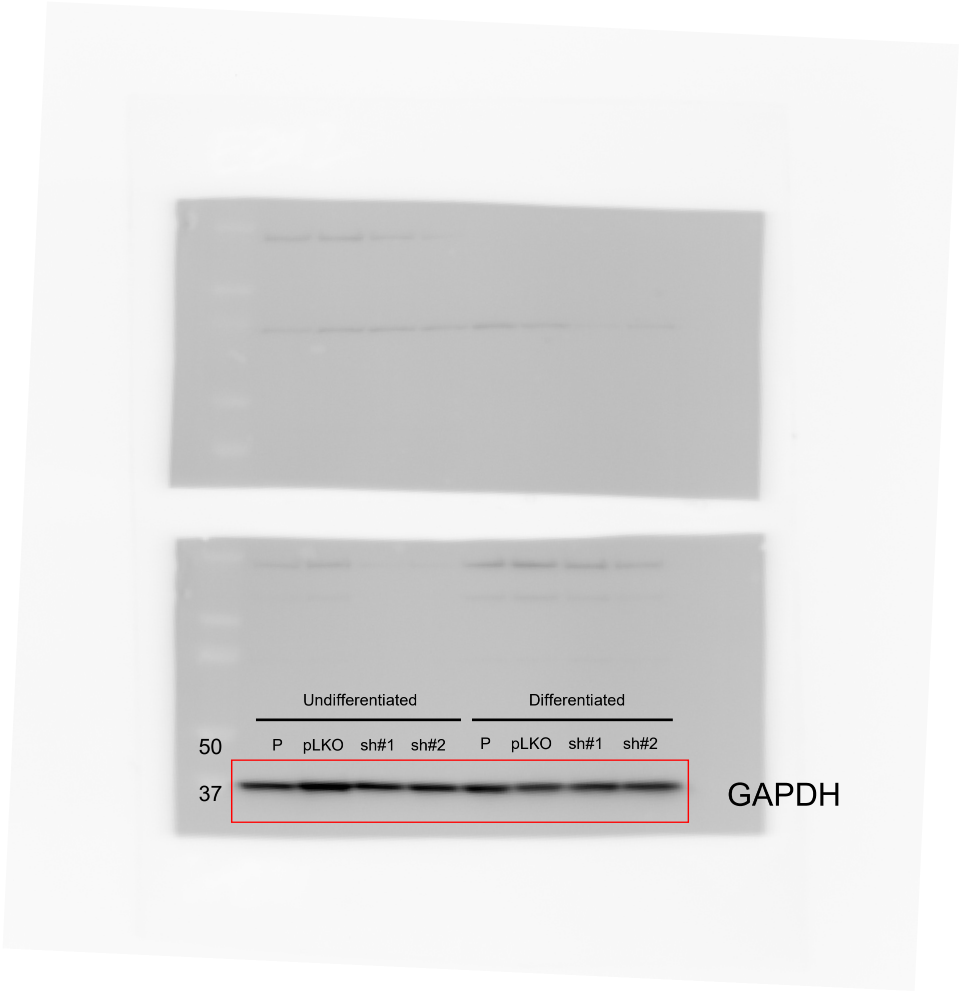

S1 Fig

Scans of Western Blots for Fig. 5B
(The region shown in the Figure is denoted by a red box)

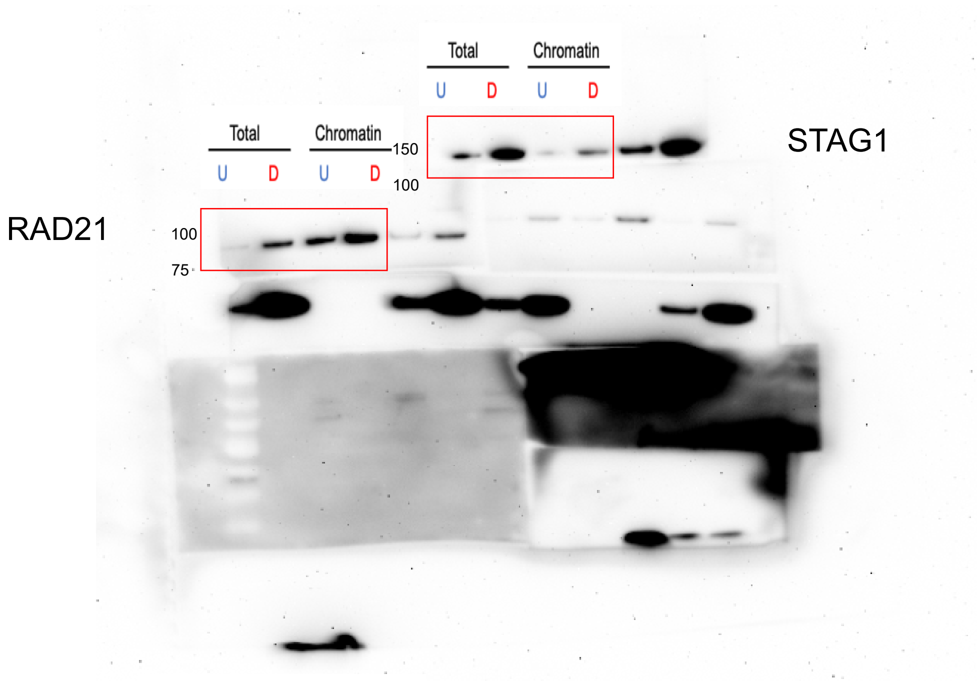

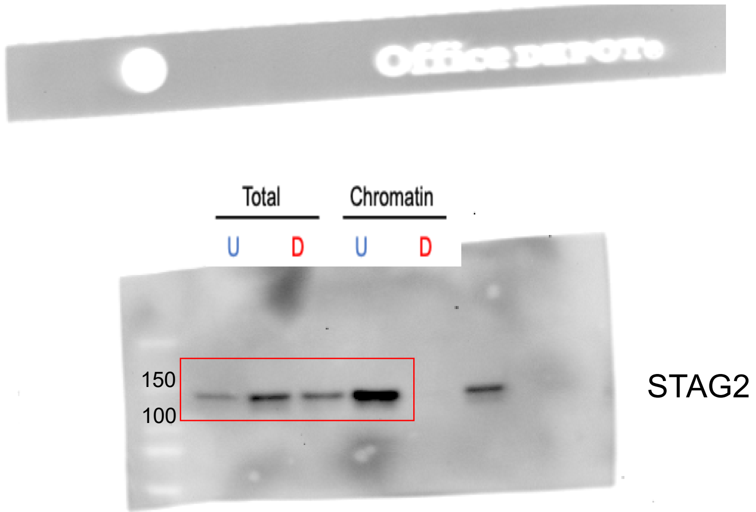

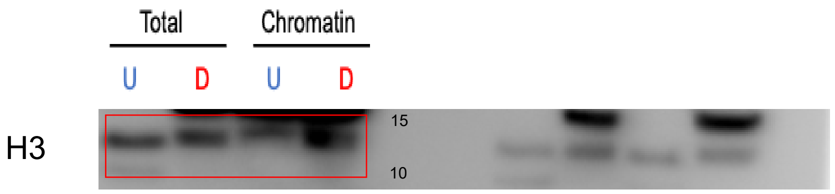

S1 Fig

Scans of Western Blots for Fig. 5B

(The region shown in the Figure is denoted by a red box)

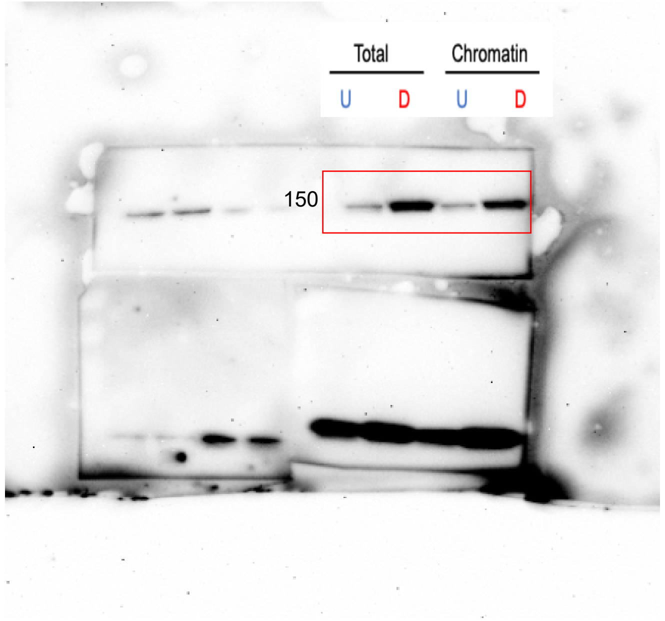

SMC1A

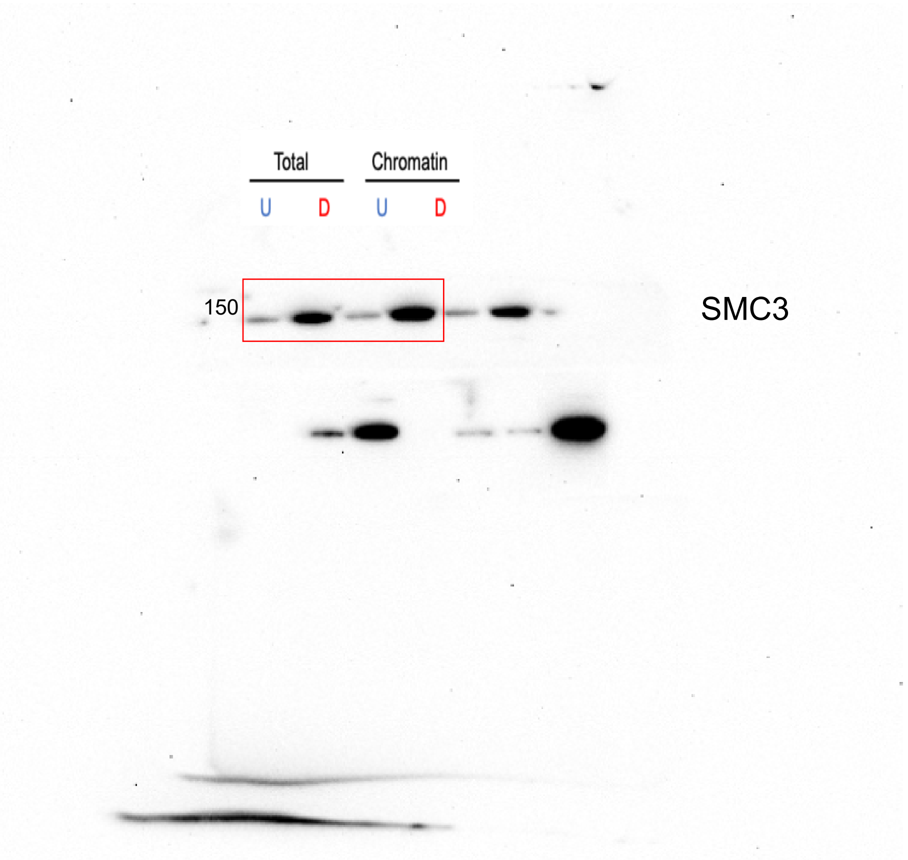

SMC3
