# Supplementary material for: STAG2 regulates polycomb and differentiation in urothelial precursors and bladder cancer
Source: PLoS One. 2025 Oct 15;20(10):e0333128. doi: 10.1371/journal.pone.0333128 (PMC12527211; doi:10.1371/journal.pone.0333128)
Supplement: S2 Fig — (PPTX) [file pone.0333128.s002.pptx]

## Slide 1
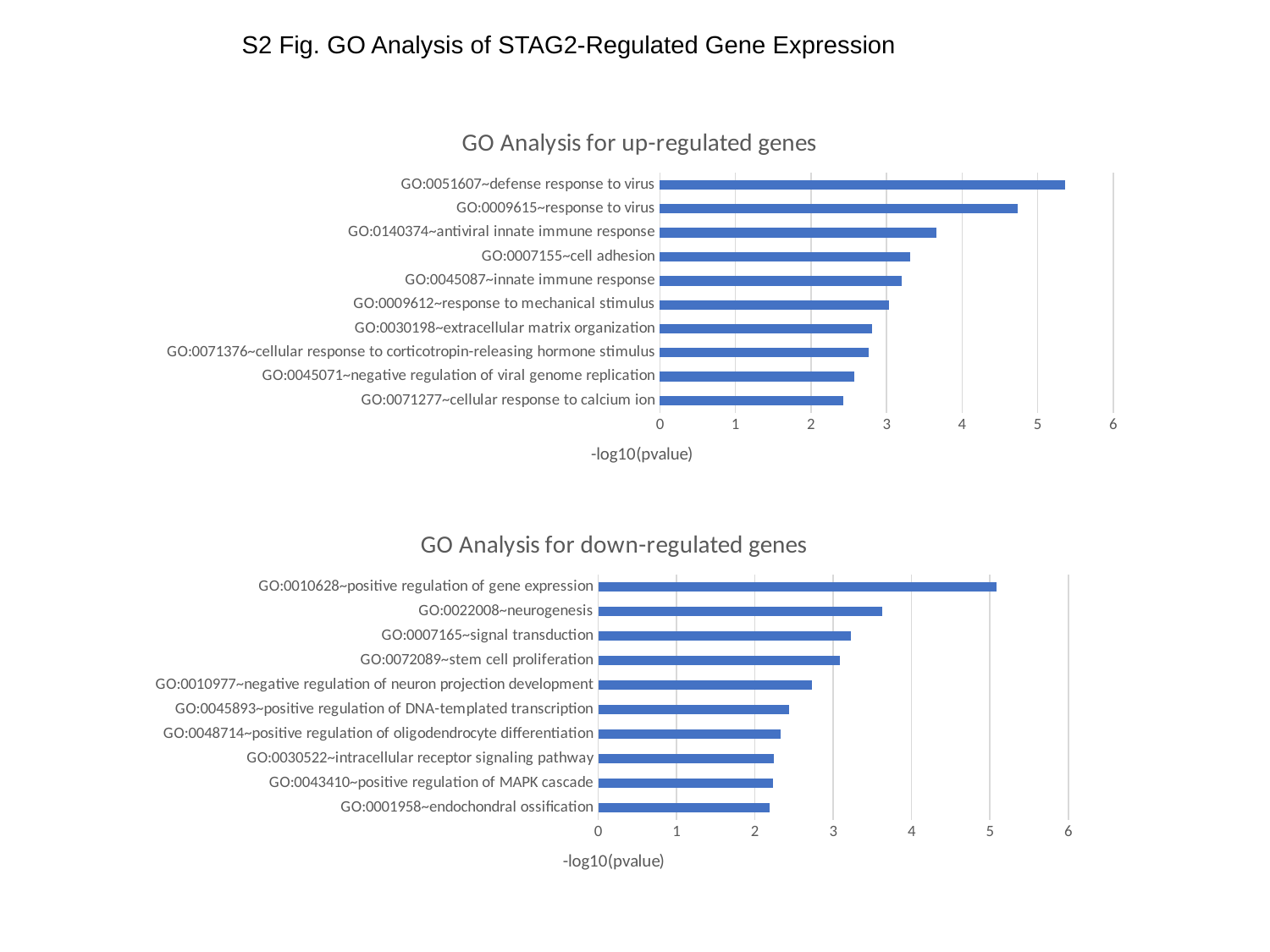

S2 Fig. GO Analysis of STAG2-Regulated Gene Expression
### Chart: GO Analysis for up-regulated genes
| Category | |
|---|---|
| GO:0071277~cellular response to calcium ion | 2.4233578083502656 |
| GO:0045071~negative regulation of viral genome replication | 2.569718037214707 |
| GO:0071376~cellular response to corticotropin-releasing hormone stimulus | 2.7689686854784723 |
| GO:0030198~extracellular matrix organization | 2.802918610663821 |
| GO:0009612~response to mechanical stimulus | 3.0284848665033492 |
| GO:0045087~innate immune response | 3.200880665849263 |
| GO:0007155~cell adhesion | 3.3089369318655955 |
| GO:0140374~antiviral innate immune response | 3.659833237857995 |
| GO:0009615~response to virus | 4.737642283000594 |
| GO:0051607~defense response to virus | 5.362756050919654 |
### Chart: GO Analysis for down-regulated genes
| Category | |
|---|---|
| GO:0001958~endochondral ossification | 2.1905306807784704 |
| GO:0043410~positive regulation of MAPK cascade | 2.2261226901306452 |
| GO:0030522~intracellular receptor signaling pathway | 2.2420328554969617 |
| GO:0048714~positive regulation of oligodendrocyte differentiation | 2.3260180812318216 |
| GO:0045893~positive regulation of DNA-templated transcription | 2.437800724173346 |
| GO:0010977~negative regulation of neuron projection development | 2.72448669272284 |
| GO:0072089~stem cell proliferation | 3.0807285861379956 |
| GO:0007165~signal transduction | 3.220976716368678 |
| GO:0022008~neurogenesis | 3.6284788173147158 |
| GO:0010628~positive regulation of gene expression | 5.087037272031854 |
