# Supplementary material for: STAG2 regulates polycomb and differentiation in urothelial precursors and bladder cancer
Source: PLoS One. 2025 Oct 15;20(10):e0333128. doi: 10.1371/journal.pone.0333128 (PMC12527211; doi:10.1371/journal.pone.0333128)
Supplement: S3 Fig — GSEA signatures meeting a threshold of FDR < 0.05 are shown. (PPTX) [file pone.0333128.s003.pptx]

## Slide 1
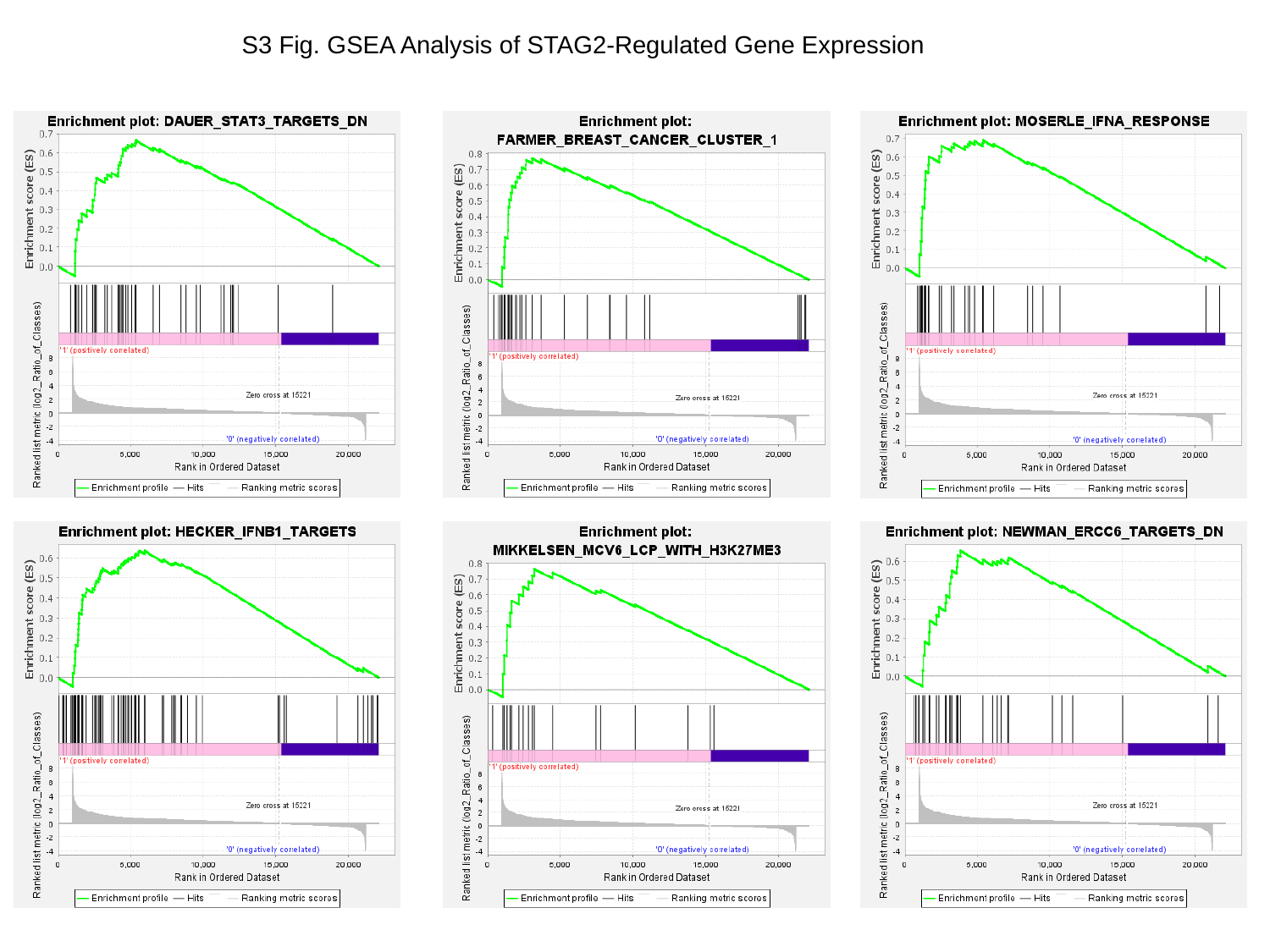

S3 Fig. GSEA Analysis of STAG2-Regulated Gene Expression

## Slide 2
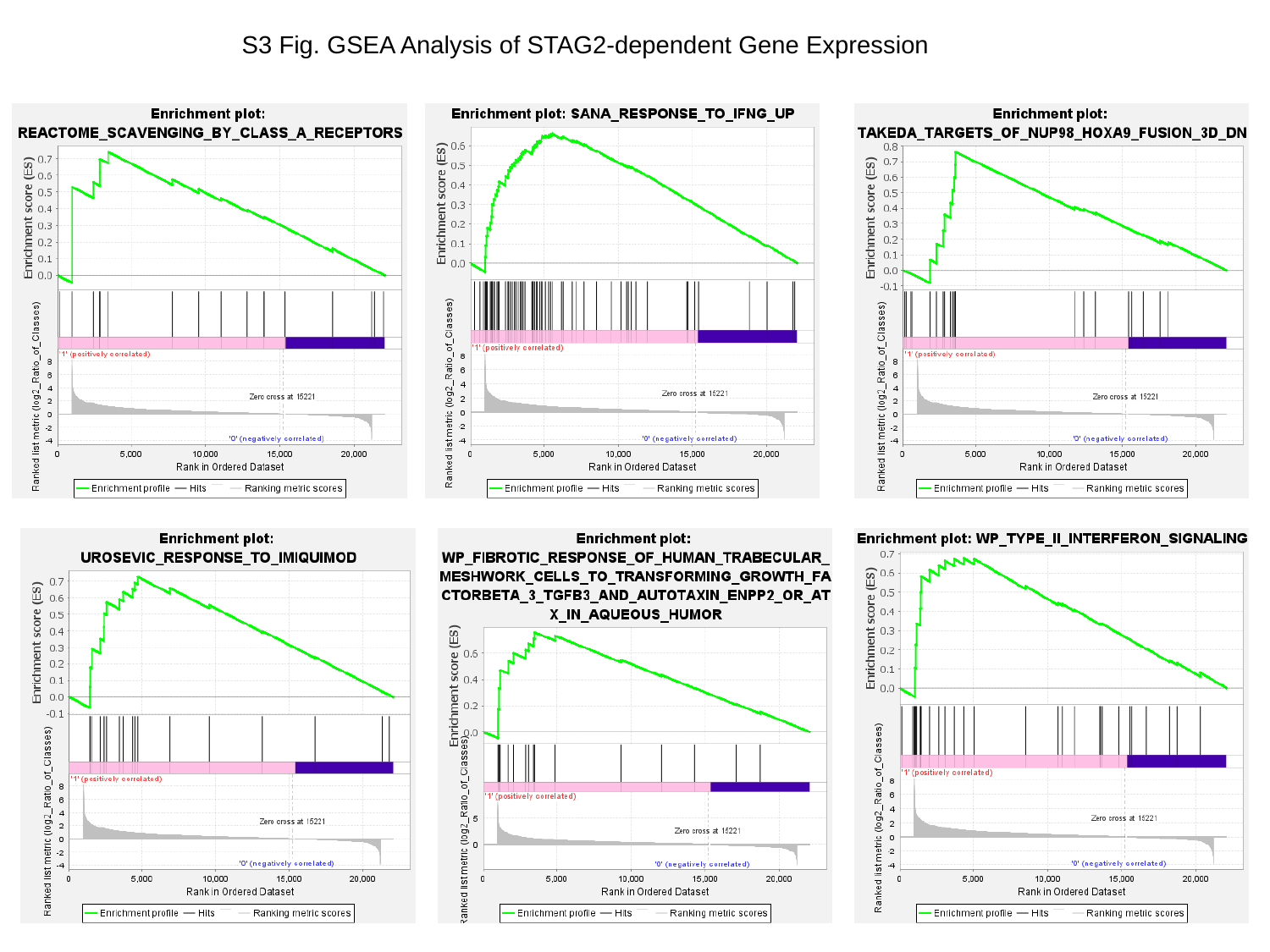

S3 Fig. GSEA Analysis of STAG2-dependent Gene Expression
