# Supplementary material for: STAG2 regulates polycomb and differentiation in urothelial precursors and bladder cancer
Source: PLoS One. 2025 Oct 15;20(10):e0333128. doi: 10.1371/journal.pone.0333128 (PMC12527211; doi:10.1371/journal.pone.0333128)
Supplement: S4 Fig — (A) Schematic of STAG2 exon 5/intron 5/exon 6. (B) Gene editing strategy for correction of the STAG2 splice acceptor mutation in VM-CUB-3 cells. The location of the splice acceptor mutation in VM-CUB-3 cells is shown with a red asterix. An AAV-based gene editing vector was generated in which ~1 kb homology arms composed of wild-type STAG2 genomic sequence flank a FLOXed IRES-neoR gene. After infection of VM-CUB-3 cells, a subset of neoR clones have integrated the gene editing vector by homologous recombination, resulting in correction of the splice acceptor mutation. The FLOXed neoR gene in intron 6 is then removed by cre-recombination by infection with a cre-expressing adenovirus. (PPTX) [file pone.0333128.s004.pptx]

## Slide 1
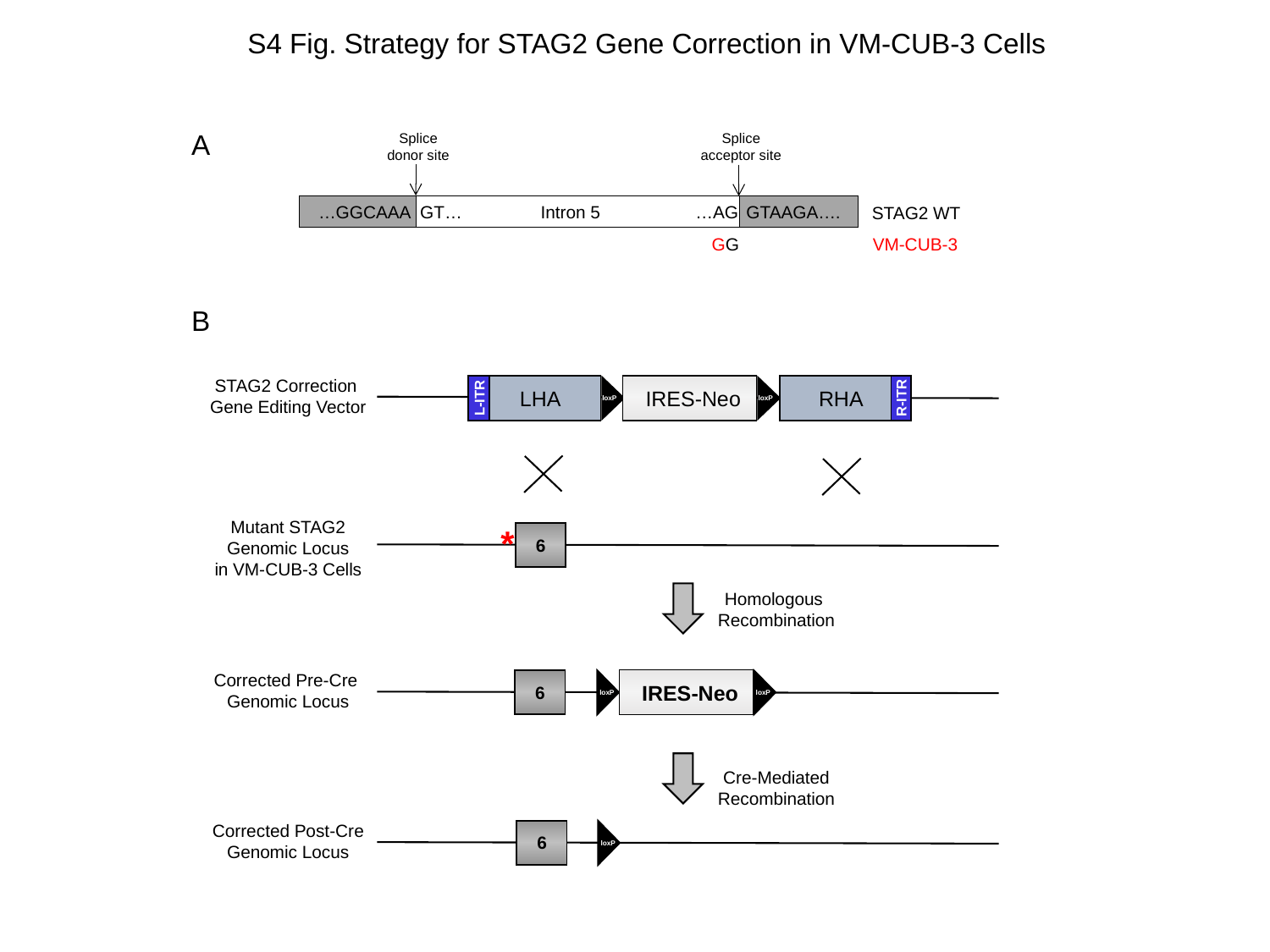

S4 Fig. Strategy for STAG2 Gene Correction in VM-CUB-3 Cells
A
Splice
donor site
Splice
acceptor site
…GGCAAA
GT…
Intron 5
…AG
GTAAGA….
STAG2 WT
GG
VM-CUB-3
B
STAG2 Correction
Gene Editing Vector
LHA
RHA
IRES-Neo
L-ITR
R-ITR
loxP
loxP
Mutant STAG2
Genomic Locus
in VM-CUB-3 Cells
*
6
Homologous
Recombination
Corrected Pre-Cre
Genomic Locus
IRES-Neo
6
loxP
loxP
Cre-Mediated Recombination
Corrected Post-Cre
Genomic Locus
6
loxP

## Slide 2
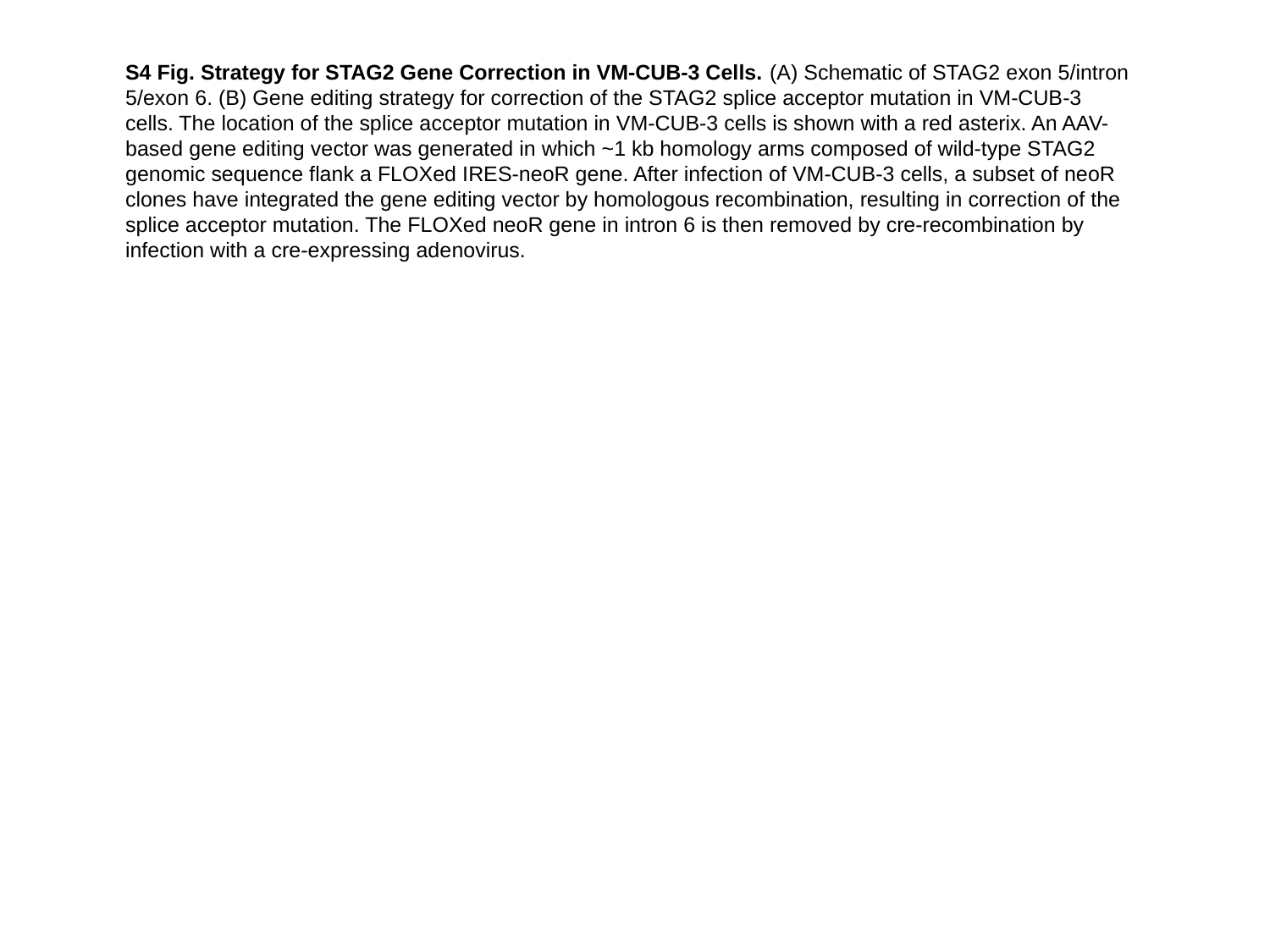

S4 Fig. Strategy for STAG2 Gene Correction in VM-CUB-3 Cells. (A) Schematic of STAG2 exon 5/intron 5/exon 6. (B) Gene editing strategy for correction of the STAG2 splice acceptor mutation in VM-CUB-3 cells. The location of the splice acceptor mutation in VM-CUB-3 cells is shown with a red asterix. An AAV-based gene editing vector was generated in which ~1 kb homology arms composed of wild-type STAG2 genomic sequence flank a FLOXed IRES-neoR gene. After infection of VM-CUB-3 cells, a subset of neoR clones have integrated the gene editing vector by homologous recombination, resulting in correction of the splice acceptor mutation. The FLOXed neoR gene in intron 6 is then removed by cre-recombination by infection with a cre-expressing adenovirus.
