# Supplementary material for: STAG2 regulates polycomb and differentiation in urothelial precursors and bladder cancer
Source: PLoS One. 2025 Oct 15;20(10):e0333128. doi: 10.1371/journal.pone.0333128 (PMC12527211; doi:10.1371/journal.pone.0333128)
Supplement: S2 Table — (DOCX) [file pone.0333128.s006.docx]

S2 Table. Taqman Assays Used in This Study

| Gene | Thermo Fisher Catalog Number | Amplicon Length |
| --- | --- | --- |
| STAG2 | Hs00198227_m1 | 82 bp |
| SCG2 | Hs01920882_s1 | 116 bp |
| ADGRF1 | Hs01587926_m1 | 127 bp |
| VCAN | Hs04978231_m1 | 97 bp |
| TNIK | Hs00323234_m1 | 65 bp |
| CPA4 | Hs00275311_m1 | 72 bp |
| NQO1 | Hs01045993_g1 | 71 bp |
| SEMA3C | Hs00989373_m1 | 71 bp |
| GALNT5 | Hs00294826_m1 | 75 bp |
| CK20 | Hs03004183_m1 | 109 bp |
| UPK2 | Hs00171854_m1 | 81 bp |
